# Trends in Rates of Opioid Agonist Treatment and Opioid-Related Deaths for Youths in Ontario, Canada, 2013-2021

**DOI:** 10.1001/jamanetworkopen.2023.21947

**Published:** 2023-07-06

**Authors:** Tea Rosic, Gillian Kolla, Pamela Leece, Sophie Kitchen, Tara Gomes

**Affiliations:** 1 Department of Psychiatry, University of Ottawa, Ottawa, Ontario, Canada; 2 Department of Health Research Methods, Evidence, and Impact, McMaster University, Hamilton, Ontario, Canada; 3 Canadian Institute for Substance Use Research, University of Victoria, Victoria, British Columbia, Canada; 4 Unity Health Toronto, Toronto, Ontario, Canada; 5 Public Health Ontario, Toronto, Ontario, Canada; 6 Department of Family and Community Medicine, University of Toronto, Toronto, Ontario, Canada; 7 Dalla Lana School of Public Health, University of Toronto, Toronto, Ontario, Canada; 8 ICES, Toronto, Ontario, Canada; 9 Leslie Dan Faculty of Pharmacy, University of Toronto, Toronto, Ontario, Canada; 10 Institute for Health Policy, Management and Evaluation, University of Toronto, Toronto, Ontario, Canada

## Abstract

**Importance:**

Data suggest that the opioid crisis in North America has recently been reflected in opioid-related mortality among youths. Despite recommendation for its use, youths encounter barriers to accessing OAT, including stigma, burden of witnessed dosing, and lack of availability of youth-oriented services and prescribers comfortable treating this population.

**Objective:**

To compare rates of opioid agonist treatment (OAT) and opioid-related mortality between youths aged 15 to 24 years and adults aged 25 to 44 years in Ontario, Canada, over time.

**Design, Setting, and Participants:**

This cross-sectional analysis of rates of OAT and opioid-related deaths between 2013 and 2021 used data obtained from the Ontario Drug Policy Research Network, Public Health Ontario, and Statistics Canada. Individuals included in the analysis were aged 15 to 44 years and residing in Ontario, the most populous province in Canada.

**Exposures:**

Youths aged 15 to 24 years compared with adults aged 25 to 44 years.

**Main Outcomes and Measures:**

OAT (methadone, buprenorphine, and slow-release oral morphine) per 1000 population and opioid-related deaths per 100 000 population.

**Results:**

Between 2013 and 2021, 1021 youths aged 15 to 24 years died from opioid toxicity; 710 were male (69.5%). In the final year of the study period, 225 youths (146 male [64.9%]) died from opioid toxicity, and 2717 (1494 male [55.0%]) were dispensed OAT. Over the study period, the rate of opioid-related deaths among youths in Ontario increased 369.2% from 2.6 to 12.2 per 100 000 population (48 to 225 total deaths) and the rate of OAT use decreased 55.9% from 3.4 to 1.5 per 1000 (6236 to 2717 individuals). For adults aged 25 to 44 years, the rate of opioid-related deaths increased 371.8% from 7.8 to 36.8 per 100 000 (283 to 1502 deaths), and the rate of OAT increased 27.8% from 7.9 to 10.1 per 1000 population (28 667 to 41 200 individuals). Trends for youths and adults persisted across both sexes.

**Conclusions and Relevance:**

The findings of this study suggest that opioid-related deaths are increasing among youths while OAT use is paradoxically declining. The reasons for these observed trends require further investigation, including a consideration of changing trends in opioid use and opioid use disorder among youths, barriers to OAT, and opportunities to optimize care and reduce harms for youths who use substances.

## Key Points

**Question:**

How have rates of opioid agonist treatment (OAT) and opioid-related deaths in youths changed over the last 9 years and how does this compare to trends among adults?

**Findings:**

This cross-sectional study of public health data for individuals aged 15 to 44 years in Ontario, Canada, found that rates of opioid-related deaths increased in both youths and adults between 2013 and 2021, yet rates of OAT among youths declined over this time despite rising rates of OAT among adults.

**Meaning:**

These results suggest that a better understanding of prevalence of opioid use disorder in youths and barriers to accessing OAT is needed.

## Teaser

This cross-sectional study of youths in Ontario, Canada, tracks changes of rates in opioid agonist treatment and opioid-related deaths from 2013 to 2021.

## Introduction

The overdose crisis across North America continues to worsen. While opioid-related deaths concentrate among middle-aged adults, recent data suggests an acceleration in opioid-related mortality among youths.^1,2^ Opioid agonist treatment (OAT) is a core response to this crisis as it is an evidence-based treatment shown to reduce opioid-related and all-cause mortality among individuals with opioid use disorder (OUD). Despite recommendation for its use,^3^ there are barriers to accessing OAT among youths, including: stigma, burden of witnessed dosing, and lack of availability of youth-oriented services and prescribers comfortable treating this population.^4,5,6^ Given the rising burden of opioid-related mortality in younger demographics, and the known barriers to accessing OAT in this population, we sought to contrast rates of OAT and opioid-related mortality between youths aged 15 and 24 years and adults aged 25 to 44 years in Ontario, Canada.

## Methods

The use of the data in this project was authorized under section 45 of Ontario’s Personal Health Information Protection Act (PHIPA) and did not require review by a research ethics board. This study followed reporting guidelines for observational trials as per the Strengthening the Reporting of Observational Studies in Epidemiology (STROBE) reporting guideline.

We conducted a repeated cross-sectional analysis of rates of OAT (methadone, buprenorphine, or slow-release oral morphine [SROM]; per 1000 population) and opioid-related deaths (per 100 000 population) between 2013 and 2021 in Ontario, the most populous province in Canada. Opioid-related deaths were obtained in aggregate form from the Public Health Ontario Interactive Opioid Tool.^7^ The Public Health Ontario Interactive Opioid tool uses data obtained by the Office of the Chief Coroner and Ontario Forensic Pathology Services.^8^ Confirmed and suspected opioid-related deaths in Ontario are investigated by this office and opioid-related deaths are defined by toxicity caused by a consumed substance where 1 or more substances was an opioid.^9^ All unexpected or unnatural deaths must be reported to the coroner for investigation of cause of death, which includes toxicologic testing, as per Canadian law.^10^ We used the Narcotics Monitoring System to capture rates of OAT using methods applied in the Ontario Drug Policy Research Network (ODPRN) Ontario Opioid Indicator Tool.^11^ The Narcotics Monitoring System was implemented in Ontario in May 2012 to track pharmacy dispensing information about all controlled drugs. OAT dispensing is monitored within this system, regardless of the payment type (eg, privately paid, public drug insurance). This data set was linked using unique encoded identifiers and analyzed at ICES. We used the Statistics Canada population estimates for Ontario by age and sex.^11^

### Statistical Analysis

We restricted the population for our primary analysis to youths aged 15 to 24 years and constructed a comparator cohort of adults aged 25 to 44 years. This comparator group was selected as the age 25 to 44 years group is the demographic with the largest burden of opioid deaths and access to treatment in Ontario. In secondary analyses, we stratified rates by sex and OAT type. Data on race and ethnicity are not available within the datasets used and therefore could not be reported. For each age-specific cohort and strata, we determined the relative percentage change from 2013 to 2021 for OAT use and opioid-related deaths. All analyses were performed in SAS Enterprise Guide version 7.1 (SAS Institute).

## Results

Between 2013 and 2021, 1021 youths aged 15 to 24 years died from opioid toxicity, the majority of whom were male (710 [69.5%]). In the final year of our study period, 225 youths (64.9% male) died from opioid toxicity, and 2717 (55.0% male) were dispensed OAT.

### Overall Rates of OAT and Opioid-Related Deaths for Youths Compared With Adults

**Figure 1.**
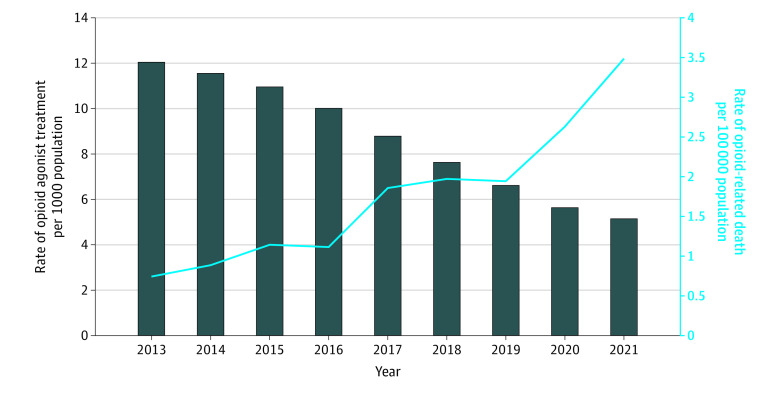
Rates of Overall Opioid Agonist Treatment and Opioid-Related Deaths Among Youths Rates of opioid-related deaths (per 100 000 population) and opioid agonist treatment (per 1000 population) in Ontario from 2013 to 2021 for youths aged 15 to 24.

**Figure 2.**
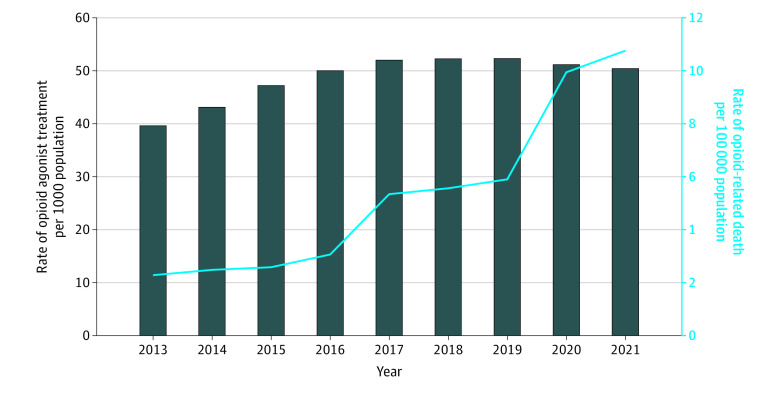
Rates of Overall Opioid Agonist Treatment and Opioid-Related Death for Adults Rates of opioid-related deaths (per 100 000 population) and opioid agonist treatment (per 1000 population) in Ontario from 2013 to 2021 for adults aged 25 to 44.

Between 2013 and 2021, the rate of opioid-related deaths among youths in Ontario increased 369.2% from 2.6 to 12.2 per 100 000 population (48 to 225 total deaths), whereas the rate of OAT decreased 55.9% from 3.4 to 1.5 per 1000 population (6236 to 2717 individuals) (Figure 1). Among adults aged 25 to 44 years, the rate of opioid-related deaths also increased (371.8% from 7.8 to 36.8 per 100 000 [283 to 1502 deaths]) between 2013 and 2021, but in contrast to trends observed among youths, the rate of OAT increased 27.8% from 7.9 to 10.1 per 1000 population (28 667 to 41 200 individuals) (Figure 2).

### Secondary Analyses

**Figure 3.**
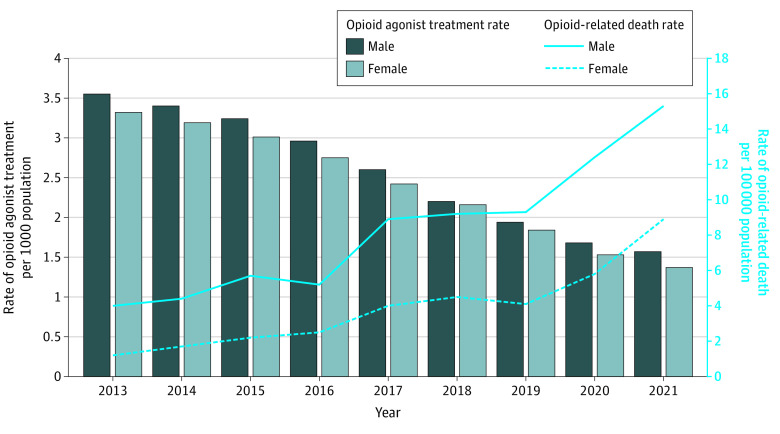
Rates of Overall Opioid Agonist Treatment and Opioid-Related Deaths for Youths, by Sex Rates of opioid-related deaths (per 100 000 population) and opioid agonist treatment (per 1000 population) in Ontario from 2013 to 2021 for youths aged 15 to 24 stratified by sex (males vs females).

The trends documented among youths were largely consistent when stratified by sex. In general, rates of OAT and opioid-related deaths were higher among males throughout the study period. By 2021, rates of OAT had declined to similar levels among males and females (1.6 vs 1.4 per 1000, respectively), but opioid-related death rates were much higher among males compared with females (15.3 vs 8.9 per 100 000 population, respectively) (Figure 3).

Trends in rates of opioid-related deaths were similar among adults aged 25 to 44 years when stratified by sex, although much higher than rates observed among youths (eFigure in Supplement 1). Rates of OAT were increasing among both male and female adults (albeit leveling off from 2017 to 2020). In 2021, the rate of OAT among males aged 25 to 44 years was 12.2 per 1000 population, compared with 7.9 per 1000 among females in this age group.

When stratified by OAT type, the rates of methadone treatment among youths declined 73.3% over the study period from 3.0 to 0.8 per 1000 population (eTable in Supplement 1). In contrast, the rate of buprenorphine treatment increased slightly between 2013 to 2018 (from 0.7 to 1.0 per 1000 population), before declining to 0.8 per 1000 population by 2021. Rates of SROM were consistently low in youths throughout the study period (range 0.01-0.10 per 1000 population). Among adults, the rate of methadone prescribing remained relatively consistent across the study period (6.9 per 1000 in 2013 vs 6.6 per 1000 in 2021), and buprenorphine use grew 200.0% between 2013 (1.4 per 1000) and 2021 (4.2 per 1000).

## Discussion

In this population-based study of youths and adults across Ontario, we found that, despite increasing rates of opioid-related deaths, rates of OAT among youths declined considerably over the 9-year study period, in direct contrast to increasing rates of treatment among adults.

The decline in use of OAT specifically among youths warrants further attention and consideration as barriers in OAT access for youths have long been identified. Particularly for adolescents, there exists ongoing hesitance among clinicians to start OAT, and youths experience significant stigma related to using OAT to treat OUD.^12,13^ For example, fewer than 25% of American youths diagnosed with OUD receive OAT (and fewer than 2% of those younger than 18 years).^5,14^ There are low rates of OAT initiation even following emergency care for opioid overdoses in this population.^6^ This is despite the recommendation for the use of OAT in youths with OUD by the American Academy of Pediatrics in 2016.^4^ Challenges in access to care are not limited to youths with OUD but exist more broadly across mental health and substance use disorders. Youths may be more reluctant to seek or engage in care due to difficulties navigating the system, fear of breaches of their confidentiality, and a preference toward self-management of symptoms.^15^ A number of strategies have been shown to improve the engagement of youths in care, including youth participation in program development, parental involvement, use of technology, and engagement of schools in screening and intervention.^16^

The observed decline in rates of OAT in Ontario youths may also reflect lower underlying rates of OUD in this population. Data on the prevalence of OUD in North American youths are lacking; however, cross-sectional survey data suggest that the prevalence of opioid use is decreasing among adolescents. The Ontario Student Drug Use and Health Survey has documented a drop in nonmedical opioid use by students in grades 7 to 12 from 20% (2007) to 10.6% (2019).^17^ Decreases in use of OAT in this population must be interpreted with caution as lower rates of opioid use and subsequent development of OUD in youths may influence OAT initiation rates.

Despite these trajectories in reported opioid use among youths, the declining rate of OAT among youths is concerning as it occurs over a time when there are rising rates of opioid-related deaths in this demographic. A key driver of the increase in opioid-related mortality since 2016 has been the increasing toxicity and unpredictability of the unregulated opioid supply, with 87% of all opioid-related deaths in Ontario in 2021 having fentanyl as a direct contributor to death.^9^ Toxic effects in the 15- to 24-year-old group may occur among youths who use drugs occasionally or who engage in polysubstance use and do not have an OUD diagnosis. This reinforces the need for urgent efforts to better understand patterns of substance use in this demographic to tailor services to the needs of youths and ensure access to OAT for youths who may benefit from it.

Trends documented in this study must be situated within the context of the COVID-19 pandemic, which led to interruptions in the delivery of health care services as a result of strained health care resources and public health restrictions aimed at reducing spread of the virus. In response to pandemic shifts in health care delivery, a number of changes in the delivery of care may have affected access to OAT. Some studies in Canada^18^ and in other jurisdictions^19^ have demonstrated increased access to OAT related to implementation of virtual care services for OUD, increase in take-home doses and home delivery of medications, and reduced urine drug screening requirements, among other measures. Some of these pandemic-related changes were also associated with lower rates of treatment interruption and discontinuation.^20^ Despite this, during the pandemic, there was a documented rising in opioid deaths in all populations. Our findings indicate that rising harms were also experienced among youths aged 15 to 24 years with rapid acceleration in opioid deaths in 2020 and 2021, but continued declines in OAT. While this study was not designed to study the specific effects of the pandemic, our results demonstrate that responses to improve OAT access and retention during the COVID-19 pandemic did not lead to measurable improvements in access to OAT in the younger demographics, with treatment rates continuing to fall.

### Limitations

This study had several limitations, which included our inability to determine rates of opioid use or OUD diagnoses among youths. We were also unable to determine whether OAT was sought out by youths or the extent of use of other substance use services. Duration on treatment could not be determined in this study; however, our measure provides an assessment of accessibility to treatment (ie, any exposure to OAT), which we believe is an instructive measurement when contrasting against harms. Data on naltrexone were not available as it is not commonly used in the treatment of OUD in Canada and in Ontario, the public drug benefit program only funds naltrexone for alcohol use disorder treatment. Finally, SROM data were not available for 2013.

## Conclusions

These findings support the need for a multifaceted effort to change the trajectory of the overdose crisis and its harmful consequences, especially among youths. Specifically, more must be learned about optimizing access to treatment (including OAT) and harm reduction services for youths who use substances.

## Notes

Supplement 1. **eFigure.** Rates of Overall Opioid Agonist Treatment and Opioid-related Deaths in Ontario from 2013 to 2021 for Adults Aged 25 to 44, by Sex **eTable.** Rates of Opioid Agonist Treatment, by Treatment Type, 1000 Population

Supplement 2.
Data Sharing Statement
