# Supplementary material for: Trends in Rates of Opioid Agonist Treatment and Opioid-Related Deaths for Youths in Ontario, Canada, 2013-2021
Source: JAMA Netw Open. 2023 Jul 6;6(7):e2321947. doi: 10.1001/jamanetworkopen.2023.21947 (PMC10326639; doi:10.1001/jamanetworkopen.2023.21947)
Supplement: Supplement 1. — eFigure. Rates of Overall Opioid Agonist Treatment and Opioid-related Deaths in Ontario from 2013 to 2021 for Adults Aged 25 to 44, by Sex eTable. Rates of Opioid Agonist Treatment, by Treatment Type, 1000 Population [file jamanetwopen-e2321947-s001.pdf]

Supplemental Online Content

Rosic T, Kolla G, Leece P, Kitchen S, Gomes T. Trends in rates of opioid agonist treatment and opioid-related deaths for youths in Ontario, Canada, 2013-2021. *JAMA Netw Open.* 2023;6(7):e2321947. doi:10.1001/jamanetworkopen.2023.21947

eFigure. Rates of Overall Opioid Agonist Treatment and Opioid-related Deaths in Ontario from 2013 to 2021 for Adults Aged 25 to 44, by Sex

eTable. Rates of Opioid Agonist Treatment, by Treatment Type, 1000 Population

This supplemental material has been provided by the authors to give readers additional information about their work.

© 2023 Rosic T et al. *JAMA Network Open.*

eFigure 1. Rates of overall opioid agonist treatment and opioid-related deaths in Ontario from 2013 to 2021 for adults aged 25 to 44, by sex.

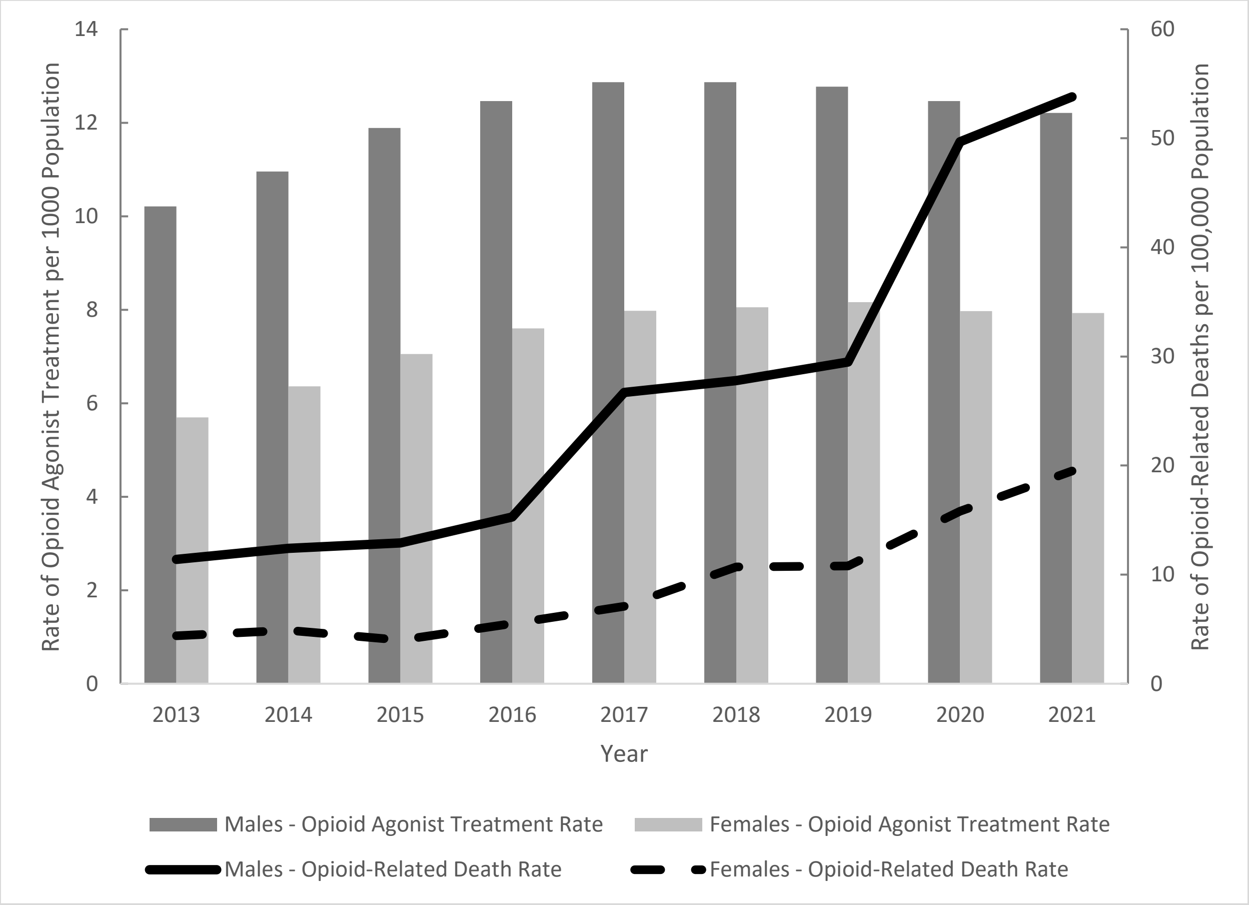

© 2023 Rosic T et al. *JAMA Network Open.*

eTable 1. Rates of opioid agonist treatment, by treatment type, 1,000 population.

	Youth aged 15-24			Adults aged 25-44		
**Year**	**Methadone**	**Buprenorphine-naloxone**[a]	**Slow-release oral morphine**[b]	**Methadone**	**Buprenorphine-naloxone**[a]	**Slow-release oral morphine**[b]
2013	2.96	0.66	-	6.89	1.37	-
2014	3.61	0.79	0.02	7.24	1.80	0.11
2015	2.43	0.91	0.02	7.61	2.32	0.09
2016	2.13	0.98	0.01	7.76	2.86	0.08
2017	1.74	1.01	0.01	7.71	3.43	0.06
2018	1.36	1.02	0.01	7.41	3.80	0.07
2019	1.10	0.99	0.01	7.13	4.23	0.10
2020	0.89	0.89	0.02	6.83	4.25	0.22
2021	0.79	0.82	0.10	6.63	4.21	0.73

[a] Buprenorphine subcutaneous formulation accounts for a small percentage of all buprenorphine treatment (< 10%) among youth and is not included in the rates provided above.
[b] Slow-release oral morphine data are unavailable in 2013.

© 2023 Rosic T et al. *JAMA Network Open.*
