# Supplementary material for: Trends in Rates of Opioid Agonist Treatment and Opioid-Related Deaths for Youths in Ontario, Canada, 2013-2021
Source: JAMA Netw Open. 2023 Jul 6;6(7):e2321947. doi: 10.1001/jamanetworkopen.2023.21947 (PMC10326639; doi:10.1001/jamanetworkopen.2023.21947)
Supplement: Supplement 2. — Data Sharing Statement [file jamanetwopen-e2321947-s002.pdf]

Data Sharing Statement

Rosic. Trends in Rates of Opioid Agonist Treatment and Opioid-related Deaths for Youths in Ontario, Canada, 2013-2021. *JAMA Netw Open*. Published July 06, 2023. doi:10.1001/jamanetworkopen.2023.21947

Data
Data available: No

Additional Information
Explanation for why data not available: Data on opioid-related harms are accessed through a public online dashboard that can be accessed by anyone without permissions. The dataset used to determine rates of OAT from this study is held securely in coded form at ICES. While legal data sharing agreements between ICES and data providers (e.g., healthcare organizations and government) prohibit ICES from making the dataset publicly available, access may be granted to those who meet pre-specified criteria for confidential access, available at www.ices.on.ca/DAS (email: das@ices.on.ca).
